# Case Report: Canakinumab for the Treatment of a Patient With COVID-19 Acute Respiratory Distress Syndrome

**DOI:** 10.3389/fimmu.2020.01942

**Published:** 2020-08-25

**Authors:** Massimo Caracciolo, Sebastiano Macheda, Demetrio Labate, Marco Tescione, Stefano La Scala, Eugenio Vadalà, Rosalba Squillaci, Francesco D’Aleo, Antonella Morabito, Cristina Garreffa, Maria Concetta Marciano, Esther N. Oliva

**Affiliations:** ^1^UOSD Terapia Intensiva Post-Operatoria, Grande Ospedale Metropolitano Bianchi Melacrino Morelli, Reggio Calabria, Italy; ^2^Intensive Care Unit, Grande Ospedale Metropolitano Bianchi Melacrino Morelli, Reggio Calabria, Italy; ^3^Dipartimento di Microbiologia e Virologia, Grande Ospedale Metropolitano Bianchi Melacrino Morelli, Reggio Calabria, Italy; ^4^Central Pharmacy, Grande Ospedale Metropolitano Bianchi Melacrino Morelli, Reggio Calabria, Italy; ^5^UOC Laboratorio Analisi, Grande Ospedale Metropolitano Bianchi Melacrino Morelli, Reggio Calabria, Italy; ^6^Hematology Unit, Grande Ospedale Metropolitano Bianchi Melacrino Morelli, Reggio Calabria, Italy

**Keywords:** COVID-19, SARS-CoV-2, canakinumab, IL-1, IL-6, cytokine storm, natural killer, acute respiratory distress syndrome

## Abstract

Severe cases of COVID-19 present with serious lung inflammation, acute respiratory distress syndrome and multiorgan damage. SARS-CoV-2 infection is associated with high cytokine levels, including interleukin-6 and certain subsets of immune cells, in particular, NK, distinguished according to the cell surface density of CD56. Cytokine levels are inversely correlated with lymphocyte count, therefore cytokine release syndrome may be an impediment to the adaptive immune response against SARS-CoV-2 infection. Canakinumab, a monoclonal antibody targeting IL-1β is under investigation for the treatment of severe SAR-CoV-2 infection. An 85 year old male presenting in our hospital with COVID-19, whose condition was complicated by acute respiratory distress syndrome and cardiac and renal failure (with oliguria) after 25 days of hospitalization, was intubated and received canakinumab for compassionate use. On the next day, diuresis recovered and conditions improved: high IL-6 levels and NK cells expressing CD56^*bright*^ (associated with cytokine relase) were significantly reduced giving rise to NK CD56^*dim*^. Patient died on day 58 with pulmonary bacterial superinfection and persistent SARS-CoV-2 positivity. In conclusion, canakinumab rescued a high risk, very elderly patient, from multiorgan damage complicating COVID-19. It may represent an useful treatment in severe cases.

## Introduction

SARS-CoV-2 is responsible for the current pandemic of coronavirus disease 2019 (COVID-19). Patients present with fever, dry cough, dyspnea, and pneumonia (1). Some patients (approximately 15%), prevalently elderly and with comorbidities, develop serious multiple organ inflammation and acute respiratory distress syndrome (ARDS) (2–4) and require intensive care unit (ICU) admission (3, 4).

The immune response, including the release of pro-inflammatory cytokines and activation of T cells, are essential for controlling the viral spread, inflammation, and tissue renewal (5, 6). The damged host cell releases proteins induce the production of pro-inflammatory cytokines by nearby cells. Monocytes, macrophages and T cells are attracted to the site of infection, establishing a pro-inflammatory feedback circuit. When the immune response is hampered, the excessive pro-inflammatory cytokines in the lungs are responsible for lung tissue damage and the cytokine storm (CS), or macrophage activation syndrome (MAS), leading to multi-organ damage.

Severe cases with CS progress to ARDS (7, 8). The mechanisms leading to such complications are complex and still under investigation. Many features of COVID-19 resemble MAS triggered by viral infection (8, 9). In fact, SARS-CoV-2 infection is associated with high levels of cytokines and, interestingly, levels of IL-6 have been reported to be correlated with mortality (10). Furthermore, in severe cases, a reduction of natural killer cells and other T lymphocytes, has been observed. Cytokine levels are inversely correlated with lymphocyte count, therefore CS may be an impediment to the adaptive immune response against SARS-CoV-2 infection (6, 11). Moreover, the severity of COVID-19 is correlated with NK subsets distinguished according to the cell surface density of CD56. In fact, CD56^*bright*^ subset increases with severity. CD56^*dim*^ NK cells physiologically comprise around 90% of NK cells in peripheral blood and are frequently described as the most cytotoxic, whereas CD56^*bright*^ NK cells are abundant cytokine producers (12, 13).

Interleukin-1 (IL-1) activates the expression of several pro-inflammatory genes. IL-1β induces inflammation during infection and autoimmunity (14). IL-1β is released by various cell types, including macrophages (15, 16). Canakinumab, a monoclonal antibody targeting IL-1β, is approved for use in rheumatologic disorders^1^. Based on the mechanism of action of canakinumab, the drug is under investigation for the treatment of severe SAR-CoV-2 infection.

We present a case of an 85 year old male presenting with COVID-19, complicated by ARDS and cardiac and renal failure, rescued by canakinumab. An indication for compassionate use for COVID-19 during the current pandemic and approval from the local ethics committee was obtained in our center.

## Case Description

**FIGURE 1:**
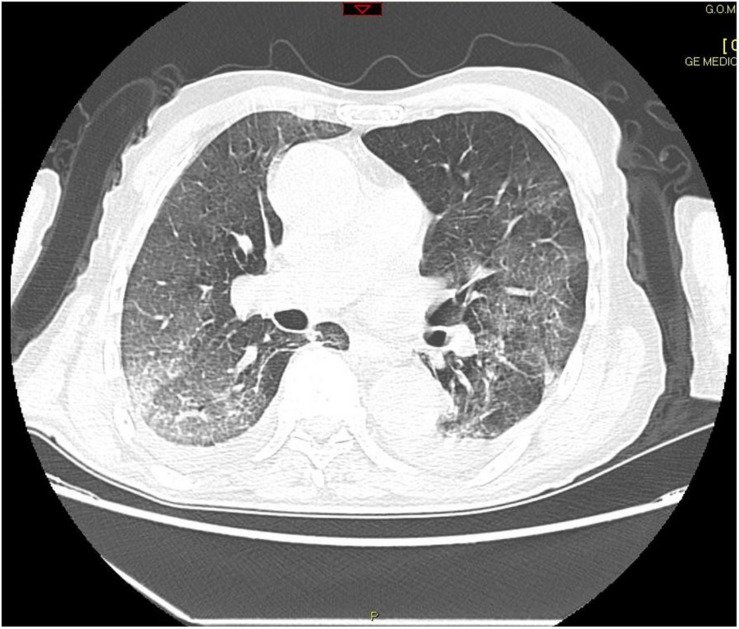
CT scan on day 3 showed bilateral, patchy alveolar opacities progressing to diffuse consolidations, with a “white lung” appearance and widespread ground-glass opacities and moderate bilateral pleural effusions.

Patient was admitted to hospital on March 23, 2020, presenting with fever (38.5°C), hypoxemia (p02 = 61 mmHg), cough, and dyspnea. Medical history revealed only mild arterial hypertension treated with amlodipine and prostatic hypertrophy not requiring treatment. SARS-CoV-2 swab was positive. Chest X-ray showed an interstitial lung pattern and small left pleural effusion. Renal and liver biochemistry were normal. Noteworthy, the patient presented lymphopenia. Reactive C Protein (RCP) was 139 mg/L. Coagulation tests were normal except for Fibrinogen 619 mg/dL and D-dimers 409 ng/mL. He was at first treated in the COVID ward and received broad spectrum antibiotics, hydroxychloroquine, and oxygen therapy with Venturi mask with 30% FiO2 setting. On day 3, a chest computerized tomography (CT) without contrast showed severe lung injury (Figure 1).

On day 4, though the fever had subsided, his respiratory condition deteriorated and continuous positive airway pressure (CPAP) non-invasive ventilation with 40% FiO2 setting and positive end-expiratory pressure (PEEP) 10 cmH_2_0 was initiated, together with azitromicin, enoxaparin sodium and lopinavir/ritonavir. On day 5, tocilizumab 8 mg/kg was administered intravenously (within a clinical trial) repeated after 12 h, while continuing hydroxychloroquine, azitromicin and enoxaparin. On day 23, his conditions precipitated with presentation of ARDS, a PaO_2_/FiO_2_ ratio (PF) of 103 (Fi0_2_ setting 60%, p0_2_ 62 mmHg) and severe arterial hypertension. He was transferred to the Intensive Care Unit (ICU) in an obnubilated and non-collaborative condition, so that he was sedated with dexmedetomidine while continuing CPAP ventilation. On day 24, patient presented oliguria with acute renal and cardiac failure and progressive respiratory failure. He was intubated and received assisted mechanical ventilation together with furosemide continuous intravenous infusion and vasopressor amines.

On day 25, the patient’s son was informed of the severity of the patient’s clinical conditions and of the risks and benefits of canakinumab treatment. He signed informed consent to administer treatment, to process and publish all relevant clinical research data and potentially identifying information. Canakinumab was administered at a single 300 mg s.c. dose on days 25 and 31.

## Diagnostic Assessment

To evaluate the biochemical effects of canakinumab, general laboratory chemistry, IL-6, and immunophenotype were collected before and after first and second administration. The drug was well tolerated in the short term, and on the day following the first administration, the patient’s diuresis normalized and renal function improved gradually without full recovery (on day 53, creatinine level reached 88 μmol/L). The findings are summarized in Table 1.

**TABLE 1:** The laboratory findings before (day 23) and after the First (day 28) and Second (day 42) administrations of canakinumab.

Variable	Before	After First	After Second
Hemoglobin (Hb) g/dL	12.0	11.3	8.7
White Blood Cell count (WBC) × 10^9^/L	4.4	6.5	12.4
Neutrophils-bands (Neutroph) × 10^9^/L	3.4	5.8	10.3
Lymphocytes (Lymph) × 10^9^/L	0.5	0.2	0.5
Platelet count (PLT) × 10^9^/L	135	107	291
D-dimer (D-d) nmol/L	2.1	1.9	3.2
Creatinine (Cr) μmol/L	44	124	97
CRP mg/L	3.1	10.2	156.0
Lactate dehydrogenase (LDH) μkat/L	5.0	3.8	3.8
Alkaline phosphatase μkat/L	1.6	1.9	1.9
Alanine aminotransferase (ALT) μkat/L	1.0	0.5	0.2
Aspartate aminotransferase (AST) μkat/L	0.5	0.4	0.4
γ-Glutamyltransferase (GGT) μkat/L	0.4	0.4	0.5
Serum IL-6, IU/ml	424.6	46.2	75.2
Immunophenotype, cells/μL			
Lymphocyte T			
CD3+	402	172	114
CD3+CD4+	309	117	74
CD3+CD8+	95	56	41
Lymphocyte B CD19+	31	32	34
Lymphocyte NK			
CD16+CD56+	111	77	18
NK CD56^*DIM*^	57	66	109
NK CD56^*BRIGHT*^	42	1	2
CD4/CD8 Ratio	3	2	2

During hospitalization, the patient underwent periodical microbiological surveillance tests. SARS-Cov-2 genome was evaluated by the Microbiology and Virology laboratory of our hospital. Samples from upper (nasopharyngeal) and lower (bronchoalveolar, bronchoaspirate, and tracheal aspirate) airways were collected and processed within 24 h. RNA-COVID 19 was evaluated using an Allplex 2019-nCoV assay that identifies three different target genes: E (envelope), RdRp (RNA-dependent RNA polymerase), and N (nucleoprotein gene). Based on the interpretation criteria, detection of one or more genes was interpreted as positive COVID-19. There was a high viral replication persisting on Day 43.

On day 31, as the respiratory conditions did not improve significantly, the Film Array Pneumonia detected the presence of bacterial infection caused by Acinetobacter C.B. Complex – 107 copies/mL – and Pseudomonas Aeruginosa – 106 copies/mL, treated initially with the association of Piperacillin + Tazobactam together with Cotrimoxazole provided intravenously QID, followed by Colistin 3000000 aerosol BID, then with Ceftazidime/Avibactam intravenous TID and finally with Doxycycline 100 mg intravenous BID (the latter ongoing).

**FIGURE 2:**
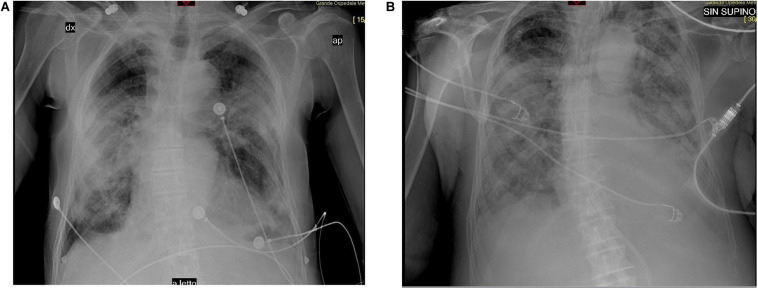
**(A)** Before the first administration of canakinumab, the chest X-ray shows interstitial changes, ground glass opacities, and multifocal and bilateral effusions. **(B)** After second administration, the chest X-ray shows the extensive bilateral opacities and bilateral effusions, concomitant to bacterial superinfection.

The initial chest CT scan on Day 13 and X-rays performed before First administration (Day 23) and after Second administration (Day 38) are shown in Figure 2.

## Discussion

Canakinumab is an IL-1 antagonist indicated to treat autoinflammatory disorders. Severe COVID-19 cases show symptoms associated with an excessive release of cytokines (17, 18).

The IL-1 cytokine family plays an important role in regulating inflammation and is produced in response to inflammatory stimuli and infections. IL-1 production requires inflammasome/Caspase-1-dependent processing. It mediates its effects by binding to its receptor to activate downstream signaling which activates MAPKs and NF-kappa B, leading to the expression of pro-inflammatory mediators that drives the IL-6 signaling pathway.

IL-6 significantly contributes to MAS. Its levels increase with the severity of COVID-19 (9, 19, 20) and the area of pulmonary infiltration (≥50%) in patients with ARDS, together with specific lymphocyte subsets (21). Though the present case had received tocilizumab prior to canakinumab, IL-6 level remained high, postulating that tocilizumab be insufficient to rescue the patient from the subsequent cytokine storm. In our patient, IL-6 and NK CD56bright both decreased after treatment with canakinumab, suggesting that canakinumab, by interfering with IL-1, reduces IL-6. Following the Second administration, IL-6 and CRP levels increased which can be explained with the development of superimposed pulmonary bacterial infection.

Several immunotherapeutic drugs are promising for the treatment of the cytokine storm associated with COVID-19 (22). Amongst these, anakinra, an IL-1 receptor antagonist, saltuximab, an IL-6 antagonist, and sarilumab, an IL-6 receptor antagonist, are undergoing Phase 3 stage development (23).

However, canakinumab treatment is associated with adverse events, mainly an increased incidence of serious infections. In fact, IL-1β physiologically contributes to host defense against infection by enhancing the antimicrobial action of phagocytes and inducing Th1 and Th17 adaptive immune responses (24). In our case, the patient survived the MAS, but developed bacterial pulmonary superinfection, which was the final cause of death on day 58. This case represents the first published report of canakinumab for the treatment of multiorgan damage associated with COVID-19.

## Patient Perspective

Excessive cytokine release induces severe complications and worsens the prognosis in COVID-19. There are numerous ongoing trials to find treatments that target virus and/or inflammation. Until an effective treatment is found, in this scenario, Canakinumab due to its blocking action of proinflammatory activity by IL-1β can constitute a potential useful treatment in the modulation of hyperinflammatory symptoms, for a subgroup of patients with COVID-19, that resemble the cytokine storm in patients with MAS. The active involvement of immunologists in the clinic and in clinical trials may improve patient outcomes.

## Ethics Statement

The studies involving human participants were reviewed and approved by Comitato Etico Sezione Sud – Regione Calabria. The patients/participants provided their written informed consent to participate in this study.

## Author Contributions

MC, EO, and DL drafted the initial manuscript. CG performed flow cytometry. FD’A performed microbiological testing. MM performed cytokine tests. All authors reviewed and revised the manuscript and have read and agreed to the published version of the manuscript to the work reported.

## Conflict of Interest

EO reports personal fees from Novartis, during the conduct of the study. The remaining authors declare that the research was conducted in the absence of any commercial or financial relationships that could be construed as a potential conflict of interest.

## Footnotes

1 https://www.drugs.com/pro/ilaris.html#s-34067-9
